# What do Cochrane systematic reviews say about diabetic retinopathy? O que as revisões sistemáticas da Cochrane dizem sobre retinopatia diabética?

**DOI:** 10.1590/1516-3180.2016.0356040117

**Published:** 2017-01-05

**Authors:** Vania Mozetic, Julia Pozzetti Daou, Ana Luiza Cabrera Martimbianco, Rachel Riera

**Affiliations:** I MD. Ophthalmologist, Dyslipidemia Service, Hospital Dante Pazzanese de Cardiologia; Postgraduate Student, Evidence-Based Health Program, Universidade Federal de São Paulo (Unifesp), São Paulo (SP), Brazil.; II Undergraduate Student of Medicine, Escola Paulista de Medicina (EPM), Universidade Federal de São Paulo (Unifesp), São Paulo (SP), Brazil.; III MSc, PhD. Physiotherapist and Research Assistant, Cochrane Brazil, São Paulo (SP), Brazil.; IV MD, MSc, PhD. Rheumatologist and Adjunct Professor, Discipline of Evidence-Based Medicine, Escola Paulista de Medicina (EPM), Universidade Federal de São Paulo (Unifesp); and Assistant Coordinator, Cochrane Brazil, São Paulo (SP), Brazil.

**Keywords:** Review [publication type], Diabetic retinopathy, Therapeutics, Evidence-based medicine, Evidence-based practice

## Abstract

**CONTEXT AND OBJECTIVE:**

Diabetic retinopathy is a disease caused by increased permeability of retinal vessels. Its incidence and prevalence have been increasing due to urbanization, greater life expectancy and the habits of modern life. Its onset is insidious and it may lead to blindness in 75% of individuals who have been diabetic for more than 20 years. The aim here was to evaluate the evidence from Cochrane systematic reviews on interventions relating to diabetic retinopathy.

**DESIGN AND SETTING:**

Review of systematic reviews, conducted at Cochrane Brazil.

**METHODS:**

We included Cochrane systematic reviews on interventions relating to diabetic retinopathy. Two researchers evaluated the inclusion criteria, summarized the reviews and presented the results narratively.

**RESULTS:**

Ten reviews met the inclusion criteria. They showed some evidence of benefits from: (a) photocoagulation for diabetic retinopathy; (b) strict glucose and pressure control for postponing the onset of retinopathy; (c) antiangiogenic drugs for macular edema (high-quality evidence); (d) anti-vascular endothelial growth factor agents for proliferative diabetic retinopathy (very low to low-quality evidence); and (e) intravitreal injection or surgical implantation for treating persistent or refractory macular edema. However, blood pressure control seems to have no benefit after the onset of retinopathy.

**CONCLUSION:**

Only a few options are likely to be effective for treating diabetic retinopathy. These include photocoagulation and anti-vascular endothelial growth factor agents. Strict glucose and pressure control seem to postpone the onset of retinopathy. For macular edema, antiangiogenic drugs, intravitreal injection and surgical implantation seem to have some benefit.

## RESUMO

**CONTEXTO:**

A retinopatia diabética é uma doença causada pelo aumento da permeabilidade dos vasos da retina. Sua incidência e prevalência vêm aumentando devido à urbanização, maior expectativa de vida e hábitos de vida modernos. Seu início é insidioso e pode levar à cegueira em 75% dos pacientes diabéticos com mais de 20 anos de doença. O objetivo foi avaliar a evidência das revisões sistemáticas Cochrane sobre intervenções para retinopatia diabética.

**TIPO DE ESTUDO E LOCAL:**

Revisão de revisões sistemáticas conduzida no Centro Cochrane do Brasil.

**MÉTODOS:**

Nós incluímos revisões sistemáticas Cochrane sobre intervenções para retinopatia diabética. Dois pesquisadores avaliaram os critérios de inclusão, resumiram as revisões e apresentaram os resultados narrativamente.

**RESULTADOS:**

Dez revisões preencheram os critérios de inclusão e mostraram benefícios com: (a) fotocoagulação para retinopatia diabética; (b) controle rigoroso da glicose e da pressão para adiar o início da retinopatia; (c) fármacos antiangiogênicos para edema macular (evidência de alta qualidade); (d) agentes antifator de crescimento do endotélio vascular para retinopatia diabética proliferativa (evidência de qualidade muito baixa a baixa); (e) injeção intravítrea ou implante cirúrgico para o tratamento do edema macular persistente ou refratário. No entanto, o controle da pressão arterial parece não ter benefício após o início da retinopatia.

**CONCLUSÃO:**

Existem poucas opções provavelmente efetivas para o tratamento da retinopatia diabética. Estas incluem fotocoagulação e agentes antifator de crescimento do endotélio vascular. O controle rigoroso da glicose e da pressão parecem adiar o início da retinopatia. Para o edema macular, fármacos antiangiogênicos, injeção intravítrea e implante cirúrgico parecem ter algum benefício.

## INTRODUCTION

Diabetic retinopathy is a secondary retinal disease caused by vascular changes due to diabetes. It is a common complication of diabetes and is the leading cause of decreased vision in the economically active population, with large negative impacts both on public health and on the social security system. It has been estimated that, because of increased life expectancy and lifestyle changes associated with urbanization, the worldwide prevalence of diabetes will rise from 126.6 million in 2010 to 191 million in 2030.^1^

According to the World Health Organization, 75% of patients with a 20-year history of type 2 diabetes have some degree of retinopathy.^2^ Nonetheless, there is still no intervention capable of preventing the emergence of retinopathy or even of preventing its progression, effectively and safely. Thus, clinical practice is limited to guidance for patients in which they are advised to maintain strict glycemic control because of the risk of disease evolution.

Like other vascular changes in diabetic patients, retinopathy starts in the endothelium. This tissue modulates vascular functions through releasing or inhibiting nitric oxide, endothelin, angiotensin and other substances that act in relation to inflammation, platelet aggregation, permeability, oxidative stress, blood clotting and vascular tone.^3^^,^^4^^,^^5^^,^^6^^,^^7^

Diabetic retinopathy is classified based on the degree of involvement of the retinal tissue and may be early non-proliferative, moderate non-proliferative, severe non-proliferative or proliferative.^8^ Early non-proliferative retinopathy is characterized by microaneurysms seen via fundoscopy; while in moderate non-proliferative (or exudative) retinopathy, it is possible to observe hard exudates. In severe non-proliferative retinopathy, in addition to the previous changes, there are soft exudates (retinal ischemia), intraretinal abnormalities (intra-microvascular retinal anomalies, IRMA) and vessels “on rosary beads”.^8^ Finally, in proliferative retinopathy, there is vascular neoformation with blood extravasation, culminating in vitreous hemorrhage. At the most advanced stage, the new vessels can lead to retinal traction with subsequent retinal detachment.^9^

Diabetic retinopathy is diagnosed through observation of the changes described above through direct and indirect fundoscopy, retinography, photographic records of the retina or angiofluor-esceinography.^8^^,^^9^ Early diagnosis is crucial for the best response to treatments, since more advanced degrees of retinopathy have worse prognoses.

Evaluations on diabetic patients without changes seen via fundoscopy or on those with early non-proliferative diabetic retinopathy need to be made annually. Those with moderate non-proliferative diabetic retinopathy need to be evaluated every six months, and those with severe non-proliferative retinopathy, every two to four months.^10^ Patients with macular edema also need to be reevaluated within six months, because if this is persistent, treatment with a macular grid is necessary in order to preserve central vision.^10^

Diabetic macular edema is a complication of diabetic retinopathy. It is defined as clinically significant macular edema when it is observed in the presence of hard exudates less than 500 µm from the center of the fovea and/or retinal edema; or if the size of the macular edema is larger than the papillary diameter (1500 µm) of the fovea, with the presence of edema, microaneurysms, soft exudates (areas of retinal ischemia) and hard exudates (lipoprotein buildups).^10^^,^^11^ The diagnosis of clinically significant macular edema is made by means of posterior pole biomicroscopy using drug-induced mydriasis.^10^^,^^11^

The practical approach most used for preventing diabetic retinopathy is strict glucose control and regular eye tracking. The therapeutic options include laser phototherapy, which includes photocoagulation and photostimulation; injection of intravitreal corticosteroids; and use of anti-vascular endothelial growth factor (VEGF) drugs (pegaptanib, ranibizumab, aflibercept and bevacizumab).

It is important to note that once macular disease has become established, treatment for diabetic retinopathy becomes essential and haste is required. On the other hand, although the therapeutic options available seem effective, they are invasive and may be associated with serious adverse events, such as visual field loss, reduced night vision, increased intraocular pressure and endophthalmitis.

Considering the global prevalence of diabetic retinopathy, its comorbidities, the consequences associated with its development and the uncertainties regarding the effectiveness and safety of the preventive and therapeutic interventions available, it is relevant to assess the current literature in order to summarize the best evidence that can guide decision-making processes relating to this important public health problem and direct future research, so as to answer questions that still remain unanswered.

## OBJECTIVES

To evaluate the evidence from Cochrane systematic reviews regarding the effectiveness and safety of interventions for prevention and treatment of diabetic retinopathy. 

## METHODS

### Design

This was a review of systematic reviews.

### Setting

This review was conducted within the Postgraduate Program on Evidence-Based Health, of the Federal University of São Paulo (Unifesp) and at Cochrane Brazil.

### Criteria for including reviews

We only included the last version of completed Cochrane systematic reviews that evaluated the effects of different interventions for preventing or treating diabetic retinopathy. The protocols of systematic reviews in progress and withdrawn reviews were not considered.

### Search for reviews

We carried out an electronic search in the Cochrane Library (via Wiley) on August 5, 2016, as presented in Table 1.

**Table 1:**
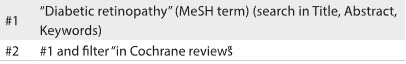
Search strategy for Cochrane Library

### Selection of reviews 

Two researchers independently selected and evaluated all the systematic reviews retrieved, in order to confirm their eligibility, in accordance with the inclusion criteria.

### Presentation of results

We presented all the included reviews narratively (qualitative synthesis). We considered that the key points regarding their relevance were the methods used, quality of studies included, results, quality of the body of final evidence for each outcome and applicability.

## RESULTS

An initial search resulted in 21 reviews and, after reading the titles and abstracts, ten Cochrane systematic reviews (SRs) were found to be actually related to the topic and fulfilled the inclusion criteria. These were then summarized and are presented below.^12^^,^^13^^,^^14^^,^^15^^,^^16^^,^^17^^,^^18^^,^^19^^,^^20^^,^^21^

### 1. Anti-vascular endothelial growth factor for prevention of postoperative vitreous cavity haemorrhage after vitrectomy for proliferative diabetic retinopathy

Vitreous hemorrhage after vitrectomy in patients with diabetic retinopathy is a major complication. In this review,^12^ the authors proposed to assess the use of anti-vascular endothelial growth factor (VEGF) after vitrectomy. Their review included randomized clinical trials (RCTs) and “quasi” randomized trials on anti-VEGF, to evaluate the incidence of vitreous hemorrhage post-vitrectomy in patients with proliferative diabetic retinopathy. Twelve RCTs of moderate quality were included, totaling 654 eyes, on patients who received bevacizumab preoperatively or intraoperatively.

Participants who received bevacizumab intravitreally, in association with vitrectomy, developed less early vitreous hemorrhage than did those who underwent vitrectomy alone. However, the effect of administering bevacizumab preoperatively or intraoperatively to prevent late vitreous hemorrhage was uncertain (risk relative, RR 0.72; 95% confidence interval, CI: 0.30 to 1.72; three studies on 196 eyes, with poor quality of evidence). No local or systemic complications were reported. The risk of retinal detachment was low among individuals who received preoperative or intraoperative treatment with bevacizumab (RR 0.46; 95% CI: 0.19 to 1.08; 7 studies on 372 participants, with low quality of evidence). The authors concluded that use of bevacizumab slowed the incidence of early vitreous hemorrhage following vitrectomy. The complications seemed few and it was believed that other ongoing studies would strengthen decision-making regarding use or nonuse of this drug.

### 2. Topical non-steroidal anti-inflammatory agents for diabetic cystoid macular edema

Cystoid diabetic macular edema, i.e. accumulation of fluid in the inner layers of the retina, is a painless complication leading to reduction or fluctuation of central vision. It may resolve spontaneously, but if it persists, it can lead to permanent loss of vision. It is probably related to inflammatory processes. Therefore, several topical non-steroidal anti-inflammatory drugs (NSAIDs), such as 0.09% bromfenac, 0.1% nepafenac and 0.5% ketorolac have been used to treat chronic diabetics with cystoid macular edema (CMO).

The aim of these authors’ review^13^ was to select randomized clinical trials and “quasi” randomized trials in order to discover the effects of topical NSAIDs among diabetics with CMO. However, no study was included, since most of the studies were conducted on pseudophakic patients. Presence of pseudophakia can be considered misleading. The authors suggested that there was a need for studies on the use of NSAIDs among diabetic patients with cystoid macular edema.

They concluded that there was a need to conduct properly designed studies in order to clarify the action of this proposed intervention on the clinical condition.

### 3. Blood pressure control for diabetic retinopathy

These authors’^14^ objective was to gather evidence regarding whether hypertension control had protective action relating to prevention and evolution of diabetic retinopathy, thereby preserving visual acuity, through measuring adverse events, quality of life and costs. Secondarily, they aimed to assess the behavior of different classes of antihypertensive drugs regarding the same outcomes. Fifteen clinical trials were included in this review, with varying follow-up times, on a total of 4,157 type 1 diabetic patients and 9,512 type 2 diabetic patients, with or without hypertension. The patients were randomized into groups with intensive pressure control versus less intensive control; standard blood pressure care versus any care; and different classes of antihypertensive drugs versus placebo.

Among type 1 diabetic patients, one out of five studies reported the incidence of diabetic retinopathy and one reported its progression over four to five years of treatment and follow-up; four studies assessed a composite outcome of incidence and progression along over the same period. Among the type II patients, five out of ten trials reported on the incidence and three reported on the progression of retinopathy; one out of these ten trials reported on both the incidence and the progression over the same time interval of four to five years. A test done among type II diabetic patients did not report the outcomes of interest for this review.

The evidence from these clinical trials showed that there was a benefit from treatment with intensive pressure control over a follow-up of four to five years, regarding the incidence of diabetic retinopathy (RR 0.8; 95% CI: 0.71 to 0.92) and the combined outcome of incidence/progression (RR 0.78; 95% CI: 0.63 to 0.97). The evidence showed that there was less benefit regarding progression over the same time interval of four to five years (RR 0.88; 95% CI: 0.73 to 1.05). Pressure control did not have any benefit regarding the progression of proliferative diabetic retinopathy, clinically significant macular edema or moderate to severe loss of visual acuity (RR 0.95; 95% CI: 0.83 to 1.09 for macular edema; and RR 1.06; 95% CI: 0.85 to 1.33 for visual acuity with the best correction), also over the same range of four to five years.

In 7 of the 15 trials, the adverse effect reported most often was death, which led to an estimated RR of 0.86 (95% CI: 0.64 to 1.14); Three trials reported hypotension as an adverse event (RR 2.08; 95% CI: 1.69 to 2.57). Ocular adverse events were described in individual trials.

In this review, the authors concluded that pressure control had a beneficial effect regarding prevention of diabetic retinopathy, but that there was no evidence that the intervention might slow down the progression of retinopathy.

### 4. Laser photocoagulation for proliferative diabetic retinopathy

Diabetic retinopathy is a complication of diabetes in which high glycemic indexes lead to damage to retinal vessels. Laser is one therapeutic option. The objective of this study^15^ was to compare laser photocoagulation with no treatment or other treatments among patients with pre-proliferative diabetic retinopathy.

These authors selected randomized clinical trials on patients with this profile and allocated them into groups of photocoagulation with any type of laser other than xenon or ruby laser. They excluded trials that compared treatments using different laser wavelengths, exposure times and powers of intensity, with absence of treatment or use of other treatments. The primary outcome was considered to be loss of three lines (15 or more letters) from visual acuity with the best correction, over two to five years. Five clinical trials totaling 4,786 people (9,503 eyes) were included in this review.

The authors took all studies with a risk of bias of execution into consideration. Three studies did not show any risk of bias due to attrition. The authors joined the data using a random effects model, except if there were three trials or fewer, in which case they used a fixed-effect model. They found that there was considerable heterogeneity among the trials, with I² greater than 50%.

In the 12^th^ month of follow-up, there was no difference between the eyes that had received photocoagulation and the eyes that had no treatment or another treatment, regarding a loss of visual acuity of 15 or more letters (RR 0.99; 95% CI: 0.89 to 1.11; two clinical trials on 8926 eyes, with low quality of evidence). Long-term follow-up did not show any consistency, but one study showed that photocoagulation reduced the risk of loss of accuracy of 15 letters or more over five years by 20%. Laser treatment reduced the risk of severe loss of visual acuity over twelve months by 50% (RR 0.46; 95% CI: 0.24 to 0.86; four clinical trials on 9,276 eyes, with moderate quality of evidence).

There was a beneficial effect on the progression of diabetic retinopathy in eyes that were treated, with a 50% reduction in the risk of progression of diabetic retinopathy (RR 0.49; 95% CI: 0.37 to 0.64; four clinical trials on 8,331 eyes, with low quality of evidence) and similar reductions in the risk of vitreous hemorrhage (RR 0.56; 95% CI: 0.37 to 0.85; two clinical trials on 224 eyes, with low quality of evidence).

The authors concluded that laser photocoagulation remained the treatment of choice for proliferative diabetic retinopathy and suggested that studies combining photocoagulation with anti-angiogenic treatment (VEGFs) should be developed.

### 5. Anti-vascular endothelial growth factor for proliferative diabetic retinopathy

Given that photocoagulation, the treatment of choice for diabetic retinopathy, has side effects of affecting the field of view and limiting night vision, the authors of this review^16^ investigated the efficiency and effectiveness of use of vascular endothelial growth factor (VEGF) as a treatment that might preserve the vision of patients with proliferative diabetic retinopathy. For this, the authors searched for randomized clinical trials comparing VEGF with sham or in combination with other treatments, among patients with proliferative diabetic retinopathy. They found 18 randomized clinical trials on a total of 1,005 patients (1,131 eyes). Eight clinical trials recruited patients referred for photocoagulation, nine for vitrectomy and one for fasciectomy, all with a mean follow-up of six months and ranges from one to twelve months. Seven studies showed a high risk of bias and the others had dubious risk of bias in one or more domains.

A study with a very low level of evidence, on 61 patients, showed that individuals treated with bevacizumab and panretinal photocoagulation were less likely to have lost three or more lines of visual acuity after 12 months, compared with those treated with panretinal photocoagulation alone (RR 0.19; 95% CI: 0.05 to 0.81). Patients treated with anti-VEGF had a higher chance of gaining three or more lines of vision acuity, but the effect was imprecise and compatible with no effect (RR 0.37; 95% CI: 6.78 to 125.95). No other study noted these two outcomes. On average, people treated with anti-VEGF (bevacizumab, ranibizumab or pegaptanib) had improved visual acuity at 12 months, compared with people who did not receive anti-VEGF (mean difference, MD -0.07; 95% CI of logarithm of the minimum angle of resolution (logMAR): -0.12 to -0.02; five clinical trials on 373 participants, with low quality of evidence). There was evidence suggesting that proliferative diabetic retinopathy regressed through reduction of leakage, seen on angiofluoresceinography, but it was difficult to estimate a result from judging only two studies. People receiving anti-VEGF were less likely to have vitreous bleeding or preretinal bleeding after 12 months (RR 0.32; 95% CI: 0.16 to 0.65; three trials on 342 participants, with low quality of evidence). No study reported health-related quality of life or fluorescein leakage.

People treated with bevacizumab and vitrectomy were less likely to lose three or more lines of vision after 12 months than were those treated with vitrectomy, but the effect was imprecise and compatible with no effect or closer to loss of vision (RR 0.49; 95% CI: 0.08 to 3.14; three trials on 94 participants, with low quality of evidence).

People treated with bevacizumab were more likely to gain three or more lines of vision (RR 1.62; 95% CI: 1.20 to 2.17; three trials on 94 participants, with low quality of evidence). In general, people treated with bevacizumab had better visual acuity after 12 months, compared with people who had not received bevacizumab, but there were doubts regarding the estimates. The confidence interval included zero, i.e. compatible with no effect, and there was considerable inconsistency between the studies (MD -0.24; 95% CI logMAR: -0.50 to 0.01; six clinical trials on 335 people, with I² = 67% and low quality of evidence). People who received bevacizumab were less likely to have pre-retinal or vitreous hemorrhage after 12 months (RR 0.30; 95% CI: 0.18 to 0.52; seven clinical trials on 393 participants, with low quality of evidence). No study reported on quality of life. Adverse effects were rarely reported and there was no evidence of any increased risk with anti-VEGF, but there were relatively few studies that reported these effects and the event occurred at a low rate. Thus, the power of analysis to detect any differences was low. The authors considered that the quality of the studies was suspect, with inaccuracy and inconsistency in assessing the risk of bias.

The authors concluded that the evidence from these clinical trials measuring the effectiveness and safety of anti-VEGF, for use in treating proliferative diabetic retinopathy to achieve standard benefits, was of low or very low quality. However, the results suggested that anti-VEGFs can reduce the risk of intraocular hemorrhage in people with proliferative diabetic retinopathy and that new clinical trials to elucidate these questions should be conducted carefully.

### 6. Anti-vascular endothelial growth factor for diabetic macular oedema

Diabetic macular edema is a common complication of diabetic retinopathy treated with grid or focal laser in order to prevent loss of vision. However, this treatment rarely improves vision. Thus, use of anti-VEGF has been proposed.

These authors^17^ investigated the effects of preserving or improving vision, acceptance, security and quality of life with this drug. They included randomized clinical trials comparing anti-VEGF drugs versus sham, other treatments or no treatment, in relation to outcomes of gain or loss of visual acuity of three or more lines, over follow-up periods of up to one year (estimated average of six months).

Eighteen studies were selected. It was concluded that over a one-year period, patients who underwent anti-VEGF treatment gained three or more lines of vision, compared with those treated using a grid (RR 3.6; 95% CI: 2.7 to 4.8; 10 trials on 1,333 cases, with high quality of evidence) and had less chance of losing three or more lines of vision (RR 0.11; 95% CI: 0.05 to 0.24; seven studies on 1,086 cases, with high quality of evidence). It was estimated that eight out of 100 patients with diabetic macular edema were able to gain three or more lines of vision by means of a macular grid, whereas 28 patients would achieve this through antiangiogenic therapy. Thus, 100 patients would need to be treated with antiangiogenic therapy in order to improve the vision of 20 patients (number need to treat, NNT = 20; 95% CI: 13-29).

People treated with anti-VEGF had an improvement of 1.6 sight lines on average (95% CI: 1.4 to 1.8) after one year, compared with those who received pan-laser photocoagulation (nine studies on 1,292 cases, with high quality of evidence). For this, seven to nine injections were applied during the first year and three or four in the second year, in larger studies, with monthly or fixed follow-up. Compared with sham treatment, the antiangiogenic was more effective (three studies on 919 participants, with high-quality evidence). Ocular adverse effects such as endophthalmitis were rare in the studies included.

A meta-analysis conducted on all the antiangiogenic drugs, compared with sham or photocoagulation, showed that there was no significant difference in relation to adverse systemic effects (15 studies with 441 events among 2985 participants; RR 0.98; 95% CI: 0.83 to 1.17), arterial thromboembolic events (14 studies with 129 events among 3034 participants; RR 0.89; 95% CI: 0.63 to 1.25) and overall mortality (63 events among 3562 participants; RR 0.88; 95% CI: 0.52 to 1.47). The authors judged that the quality of evidence regarding side effects was moderate because the safety scores were only modest and because participants with prior cardiovascular events had been excluded in some studies.

The authors concluded that there was high-quality evidence favoring use of antiangiogenic drugs, compared with photocoagulation, over a period of one to two years. They suggested that future studies should examine the real-world differences in effectiveness between the drugs used in studies monitoring patients at high cardiovascular risk.

### 7. Intensive glucose control versus conventional glucose control for type 1 diabetes mellitus

In this review,^18^ the authors analyzed the effects of strict glucose control versus conventional control, and evaluated whether blood glucose at below normal or at normal levels brought benefits. A search for randomized trials on type I diabetics with follow-ups of at least one year that had been published up to 2012 was conducted. Twelve clinical trials were found, with a total of 2,230 patients with a broad-spectrum population, with follow-ups varying from one to six and a half years. Because of the nature of the intervention, these studies could not be “blinded” to hypoglycemia. Moreover, 50% of these studies were judged to present high risk of bias in at least one other category.

In the group with strict glucose control, the risk of developing microvascular complications was lower than in the group with conventional treatment: 23/371 (6.2%) versus 92/397 (23.2%); RR 0.27; 95% CI: 0.18 to 0.42; P < 0.00001; two clinical trials on 768 participants, with high quality of evidence. Regarding the progression of the disease manifested in cases of retinopathy, the effect was weaker. For retinopathy, intensive glucose control reduced the risk of progression in studies with a duration of follow-up of at least two years: 85/366 (23.2%) versus 154/398 (38.7%); RR 0.61; 95% CI: 0.49 to 0.76; P < 0.0001; two trials on 764 participants, with moderate quality of evidence. On the other hand, there was evidence for an initial worsening of retinopathy after only one year of intensive glucose control: 17/49 (34.7%) versus 7/47 (14.9%); RR 2.32; 95% CI: 1.16 to 4.63; P = 0.02; two trials on 96 participants, with low quality of evidence).

Strict control increased the risk of hypoglycemia. However, the studies were heterogeneous, and only one study, the “Diabetes Complications Clinical Trial (DCCT)”, clearly showed any increase in episodes of severe hypoglycemia. Mortality was very low in all the studies.

### 8. Pentoxifylline for diabetic retinopathy

Vascular occlusion is a leading cause of diabetic retinopathy, since chronic high glucose levels leads to changes in the vascular endothelium that culminate in arteriolar occlusion and poor retinal tissue perfusion, rather than nourishment of these ischemic areas though stimulation from vascular proliferation factors. Pentoxifylline is a drug used in treating occlusive peripheral arterial diseases. Thus, there are clinical trials in the literature that address this subject. However, the authors of this systematic review^19^ failed to include any study in their review because none of them met the inclusion criteria proposed in their protocol.

These authors concluded that photocoagulation remained the first choice for treating diabetic retinopathy. However, there was evidence that pentoxifylline would induce decreased proteinuria and albumin excretion, and would also normalize some blood patterns. Diabetic patients treated with pentoxifylline had early absorption of retinal hemorrhage and had less neovascularization. In some cases, there was a reduction of ischemic areas. These results suggested that pentoxifylline might be effective in preventing retinal neovascularization and improving this condition. The authors suggested that further randomized clinical trials should be conducted to assess the treatment. These would be needed in order to prove the efficacy and effectiveness of pentoxifylline in relation to the evolution of diabetic retinopathy.

### 9. Vitamin C and superoxide dismutase (SOD) for diabetic retinopathy

This Cochrane review aimed to study the effects of vitamin C and superoxide dismutase (SOD), as antioxidants for treating diabetic retinopathy, given the growing evidence of the oxidizing action of this disease. The authors^20^ only took clinical trials with one or both drugs into consideration. No studies that assessed treatment of diabetic retinopathy with vitamin C and SOD to indicate whether these substances had any impact on the evolution of the disease were found.

The authors stated that photocoagulation remained the treatment of choice for diabetic retinopathy, although there was evidence that free radicals had a role in the pathogenesis of the disease. They considered that antioxidant therapy could be helpful in preventing the progression of retinopathy, and that a combination of drugs could be needed in order to prevent visual loss among diabetic patients.

### 10. Intravitreal steroids for macular edema in diabetes

In this study,^21^ the authors evaluated the safety and effectiveness of any form of steroids applied intravitreally to treat diabetic macular edema up to 2007. Seven studies on 632 eyes were included. Four studies reported on intravitreal injection of triamcinolone (IVTA), compared with other treatments, by assessing visual acuity after three, six, nine and 24 months. They showed that intravitreal steroids were more beneficial. Three studies examined intravitreal application of fluocinolone acetate implants (FAI) or systemic administration of dexamethasone (DDS). Two studies presented low risk of bias, one had medium risk, two had high risk and two had unclear risk. The results suggested that IVTA had a major beneficial effect regarding both visual acuity and retinal thickness. Two trials reported that clinical improvements were achieved through FAI, in comparison with the standard treatment, although severely decreased visual acuity was not unusual. Beneficial effects were also observed in a study using DDS, although endophthalmitis was observed and two patients presented ptosis: one with a conjunctival ulcer and one with retinal detachment. Increased intraocular pressure and cataract formation are side effects that require monitoring.

These authors concluded that intravitreal injectable steroids or implantable steroids improved visual acuity in cases of diabetic macular edema that were persistent or recurrent. However, they stated that the question of whether the same beneficial behavior in the early stages of the disease would be obtained, both with their use alone and in association with photocoagulation, remained open.

Treatment with DDS can have positive effects in cases of refractory persistent edema or in cases in which the standard treatment was insufficient. However, because of the variety of protocols, it has not been possible to identify an algorithm for its use in practice. Given that the half-life of DDS is short, patients need to be subjected to repeated injections, which increases the risk of complications relating to the procedure, such as endophthalmitis, retinal detachment and vitreous hemorrhage. FAI can solve the problem of complications due to injections, through having a more sustained effect, but it has higher risk of increased intraocular pressure, which would require medical or surgical intervention, in addition to greater risk of development of cataracts. No studies have addressed the effects of treatment according to diabetic macular edema stage, either as single or as combined therapy.

## DISCUSSION

Among the ten SRs found in the Cochrane Library that discuss interventions relating to diabetic retinopathy, four present systemic strategies that might have a preventive nature, such that they might prevent progression of the disease. These strategies would have the capacity to act throughout the microcirculation. The other six SRs analyzed local treatments for disease that had already become established.

It can be noted that among the four SRs presenting systemic interventions, “Blood pressure control for diabetic retinopathy” and “Intensive glucose control versus conventional glucose control for type 1 diabetes mellitus” were the ones that addressed prevention and progression of diabetic retinopathy. In the other two, “Vitamin C and superoxide dismutase (SOD) for diabetic retinopathy” and “Pentoxifylline for diabetic retinopathy”, the authors were unable to find relevant clinical trials and, in accordance with their predefined inclusion criteria, they left the topic open for future clinical trials, thereby revealing the need to study these issues.

The SR on the systemic intervention “Blood Pressure control for diabetic retinopathy” showed that there was a benefit from lowering blood pressure in relation to prevention of diabetic retinopathy that lasted for four or five years. However, it lacked evidence to show that this would slow the progression of diabetic retinopathy. This, together with the modest beneficial effect on disease incidence, weakened the conclusion that there was a benefit from intervening in blood pressure only to prevent diabetic retinopathy. In the review “Intensive glucose control versus conventional glucose control for type 1 diabetes mellitus”, there was high-quality evidence showing that strict glycemic control decreased the development of retinopathy complications, compared with standard control among young patients. However, the evidence relating to disease progression was weaker. These authors suggested that studies addressing the same outcomes among elderly patients with this disease and macrovascular complications should be conducted.

Systemic interventions, by their very nature, may be the most appropriate form of prevention for retinopathy. It is clear that there is a need for more studies with higher levels of evidence on prophylactic action through the microcirculation, and even on prevention relating to diabetic macrocirculation. These studies should be conducted not only on different populations, as suggested by the authors of several of the abovementioned reviews, but also on other pharmacological classes that act preventively. For example, lipid-lowering drugs are known to protect the macrocirculation, but their behavior in relation to the micro-circulation remains a mystery.

Among the six SRs that investigated local therapy, three addressed anti-VEGFs: two of these reviews analyzed studies on proliferative diabetic retinopathy and one, macular edema. One review examined clinical trials involving topical corticosteroid therapy for diabetic macular edema, and another assessed the use of non-steroidal anti-inflammatory drugs to treat cystoid macular edema. The last of these reviews examined clinical trials on photocoagulation.

In the SR “Topical non-steroidal anti-inflammatory agents for diabetic cystoid macular oedema”, the authors did not include any clinical trials that might address the use of non-steroidal anti-inflammatory drugs for treating cystoid macular edema. This was because all the studies eventually fell within the exclusion criteria due to the large number of confounding factors relating to the different etiologies of this pathological condition.

The SR “Laser photocoagulation for proliferative diabetic retinopathy” included five trials that did not address near vision or quality of life among the patients who received this treatment. It found that there was little difference in visual acuity between the control group and intervention group after a twelve-month period, with low quality of evidence. There was moderate quality of evidence regarding reduction of the risk of severe loss of visual acuity. There was a benefit regarding progression of diabetic retinopathy in the intervention group, with low quality of evidence, and also a benefit regarding vitreous hemorrhage.

In the SR “Anti-vascular endothelial growth factor for proliferative diabetic retinopathy”, the authors concluded that there was low or very low quality of evidence regarding the safety and efficacy of the use of anti-VEGFs in relation to proliferative diabetic retinopathy. However, they suggested that an improvement was obtained regarding vitreous hemorrhage. This went against the conclusion from the review “Anti-endothelial vascular growth factor for prevention of postoperative vitreous cavity haemorrhage after vitrectomy for proliferative diabetic retinopathy”, which was a review with high-quality evidence.

Among these six SRs, many concluded that the procedure investigated was advantageous. On the other hand, they suggested that further studies should be conducted on patients presenting different profiles or at earlier stages of the disease, or using associations between the therapies to enhance their effectiveness and reduce the side effects foreseen in the procedures.

Regarding visual acuity, the use of anti-VEGF in treating proliferative retinopathy was found to improve visual acuity, with low quality of evidence. There was high-quality evidence regarding its use in macular treatment, compared with use of a macular grid.

As stated earlier, diabetic retinopathy is a disease that causes a negative impact on both health and social security through affecting the economically active population. It also affects patients’ self-esteem, because of its deleterious and mutilating nature.

The treatment of choice for proliferative diabetic retinopathy continues to be peripheral retinal photocoagulation. However for treating macular disease, the use of injectable corticosteroids and anti-VEGFs is of great interest with regard to preserving and improving patients’ vision. These methods are promising alternatives for treating diabetic macular edema, but further studies on the early phase of this pathological condition are required.

Regarding the implications of the present review for further research, the need for a prophylactic treatment or an option capable of at least reducing the progression of diabetic retinopathy persists even today. The aim of such treatment would be to avoid local treatments, thereby preserving the retinal tissue. Thus, the search for systemic medication that can produce effects on the entire vascular endothelium continues, with the aim of safeguarding diabetic patients’ macro and microcirculation and acting as prophylaxis to avoid all the sequelae that diabetes causes to the vascular tree.

## CONCLUSION

Only a few options are likely to be effective for treating diabetic retinopathy. These include photocoagulation and anti-vascular endothelial growth factor agents. Strict glucose and pressure control seem to postpone the onset of retinopathy. For macular edema, antiangiogenic drugs, intravitreal injection and surgical implantation seem to have some benefit. However, these findings came from evidence ranging from low to high quality. Low-quality evidence needs to be used with caution in clinical practice until further studies can corroborate it.
